# LET’S TALK TECH: NAVIGATING TECHNOLOGY USE DECISIONS AND AN INDIRECT ROUTE TO MECHANISTIC RESEARCH

**DOI:** 10.1093/geroni/igae098.0340

**Published:** 2024-12-31

**Authors:** Clara Berridge

**Affiliations:** University of Washington, Seattle, Washington, United States

## Abstract

Grounded in the Theory of Dyadic Illness Management, Let’s Talk Tech is the first tool to support informed, shared decision making about a range of technologies commonly used in dementia care. This presentation will describe the non-linear pathway taken to incorporate SOBC to examine the mechanisms and outcomes of Let’s Talk Tech. In a Stage 1A pilot with people living with mild Alzheimer’s disease (AD) and care partners, 88% of recruited dyads (n=29 dyads) completed 100% of the self-administered intervention delivered as a web-application. This pilot established promising preliminary feasibility and efficacy on post-test outcomes, with statistically significant improvements in care partner preparedness to make decisions about technology use, knowledge of the person living with AD’s preferences, alignment, mutual understanding, and understanding of each featured technology category, as well as improvements for two categories for people living with AD. Strong interview themes clarified person-centered outcomes and surfaced additional potential mechanisms of change. By completing the intervention, all dyads developed a documented summary of the person living with AD’s choices, available for future reference and updating. Participants wanted to share these choices beyond the dyads to prevent familial conflict and obtain technology use support from their broader care networks. A Stage 1A study through the Penn AITC enabled enhancements responsive to this feedback, including electronic person-directed sharing of the documented choices using FHIR and the SMART Health Links protocol. A current Stage1B study is powered to evaluate the impact of LTT on an expanded set of hypothesized mechanisms and outcomes.

